# Xenogeneic Acellular Conjunctiva Matrix as a Scaffold of Tissue-Engineered Corneal Epithelium

**DOI:** 10.1371/journal.pone.0111846

**Published:** 2014-11-06

**Authors:** Haifeng Zhao, Mingli Qu, Yao Wang, Zhenyu Wang, Weiyun Shi

**Affiliations:** 1 Ophthalmology Department, Yijishan Hospital of Wannan Medical College, Wuhu, Anhui, China; 2 State Key Laboratory Cultivation Base, Shandong Provincial Key Laboratory of Ophthalmology, Shandong Eye Institute, Shandong Academy of Medical Sciences, Qingdao, Shandong, China; University of California, Merced, United States of America

## Abstract

Amniotic membrane-based tissue-engineered corneal epithelium has been widely used in the reconstruction of the ocular surface. However, it often degrades too early to ensure the success of the transplanted corneal epithelium when treating patients with severe ocular surface disorders. In the present study, we investigated the preparation of xenogeneic acellular conjunctiva matrix (aCM) and evaluated its efficacy and safety as a scaffold of tissue-engineered corneal epithelium. Native porcine conjunctiva was decellularized with 0.1% sodium dodecyl sulfate (SDS) for 12 h at 37°C and sterilized via γ-irradiation. Compared with native conjunctiva, more than 92% of the DNA was removed, and more than 90% of the extracellular matrix components (glycosaminoglycan and collagen) remained after the decellularization treatment. Compared with denuded amniotic membrane (dAM), the aCM possessed favorable optical transmittance, tensile strength, stability and biocompatibility as well as stronger resistance to degradation both in vitro and in vivo. The corneal epithelial cells seeded on aCM formed a multilayered epithelial structure and endured longer than did those on dAM. The aCM-based tissue-engineered corneal epithelium was more effective in the reconstruction of the ocular surface in rabbits with limbal stem cell deficiency. These findings support the application of xenogeneic acellular conjunctiva matrix as a scaffold for reconstructing the ocular surface.

## Introduction

Corneal blindness accounts for nearly 10 million cases of vision loss worldwide [1]. Keratoplasty may be the most effective therapy for corneal blindness, but the severe shortage of corneal grafts hinders the widespread application of this technique, particularly in developing countries [1], [2]. In recent years, the development of tissue engineering techniques has facilitated many investigations aimed at producing corneal cell sheets for corneal epithelium or endothelium repair. The scaffold materials include amniotic membrane collagen, fibrin and other synthetic polymers [3]–[5]. However, these carriers have exhibited defects in biocompatibility, tensile strength, stability and transparency when used as a scaffold for the tissue engineering of a cornea [6]–[11]. Human amniotic membrane from the innermost layer of the placental membranes has been widely used as a general scaffold in many ophthalmological surgical procedures. Human amniotic membrane has been used to treat numerous ocular surface disorders, including chemical and thermal burns, pterygium and persistent corneal ulcers, and to treat Stevens-Johnson syndrome and other conditions associated with limbal stem cell deficiency (LSCD) [12]. However, previous reports have shown that human amniotic membrane often degrades so quickly that it causes the failure of tissue-engineered corneal epithelium in clinical applications. This is especially true in the treatment of patients with severe ocular disorders [13]. Therefore, there is an urgent need to develop a novel ideal carrier for tissue-engineered corneal epithelium that can be used to treat patients with severe injury to the ocular surface.

The conjunctiva is a transparent mucous membrane that lines the inner surfaces of the eyelids and extends to the front surface of the eyeball, excluding the cornea. This tissue consists of non-keratinizing squamous epithelium and an underlying basement membrane. The entire conjunctiva is composed of palpebral, bulbar, and fornix regions. The bulbar conjunctiva is the thinnest and most movable part, sliding easily back and forth over the front of the eyeball. Destruction of the limbal region (site of limbal stem cells) via infection, injury, or autoimmune disease frequently results in the conjunctivalization of the corneal surface. Furthermore, both corneal and conjunctival epithelia are derived from the epidermal ectoderm. Based on the high degree of similarity between conjunctiva and cornea compared with amniotic membrane, we hypothesize that replacing the amniotic membrane with the acellular conjunctiva matrix (aCM) may be more suitable for the therapy of patients with severe ocular damage. In the current study, we present a method for preparing xenogeneic acellular conjunctiva matrix and evaluate the efficacy and safety of using it as a scaffold for tissue-engineered corneal epithelium applied to ocular surface reconstruction.

## Materials and Methods

### 2.1 Animals

Whole porcine eyes were obtained from Hengshengyuan, Inc. (Qingdao, China) and subjected to decellularization within 3 h postmortem. Male New Zealand white rabbits (weighing 1.5∼2 kg, aged 2∼4 months, Shandong Agricultural Sciences Academy, Shandong, China) were used for the isolation and culture of limbal epithelial cells and as recipients of tissue-engineered corneal epithelium transplantation. All animal experiments were performed under the Association for Research in Vision and Ophthalmology (ARVO) guidelines concerning the use of animals in ophthalmic and vision research. The protocol was approved by the Animal Care and Use Committee on the Ethics of the Shandong Eye Institute (Permit Number: SEIRB-2010-117A21). All surgery was performed under sodium pentobarbital anesthesia, and all efforts were made to minimize suffering.

### 2.2 Preparation of acellular conjunctiva matrix

Porcine conjunctiva were dissected and washed with sterile phosphate-buffered saline (PBS) containing antibiotics (100 U/ml penicillin, 100 mg/ml streptomycin) and antimycotics (2.5 µg/ml amphotericin B). After the removal of Tenon's capsule, the conjunctiva were immersed in 0.1% (w/v) SDS for 12 h at 37°C on an orbital shaker, rinsed for 10 minutes three times with PBS and dried by ventilation on a clean bench. The prepared aCM was sealed in a plastic envelope, sterilized by γ-irradiation (20 kGy), and stored at 4°C before use.

### 2.3 Evaluation of decellularization efficiency

The prepared aCM was examined by H.E. and Hochest (Santa Cruz Biotechnology, Santa Cruz, CA) staining. The remnant DNA content of prepared aCM was examined using a DNA assay kit (Biomed, Beijing, China) according to the manufacturer's instructions and detected via ultraviolet spectrophotometry (Eppendorf, Hamburg, Germany). The levels of glycosaminoglycan (GAG) and collagen were examined as in previous reports [14]. The native intact conjunctiva was used as control tissue. The ultrastructure of the aCM was examined as described previously and subsequently observed via transmission electron microscopy (TEM) (Hitachi, Tokyo, Japan) [15], [16]. The α-gal antigen was detected via immunohistochemistry using Bandeiraea simplicifolia I Isolectin B4 (BSI-B4, Sigma, St. Louis, MO). After being treated with 3% H_2_O_2_ for 30 min and 5% BSA for 15 min, frozen sections of aCM were incubated with BSI-B4 for 60 min at 37°C and then with HRP conjugated streptavidin for 60 min at room temperature. They were visualized using a DAB kit and stained with hematoxylin [17].

### 2.4 Physical analysis and stability evaluation

The thickness, optical transmittance and tensile strength of aCM were examined under wet conditions, and the values were compared with those of denuded amniotic membrane (dAM). The thickness (n = 5) was measured via Fourier-domain optical coherence tomography (FD-OCT, Optovue, Fremont, CA). Optical transmittance of each sample (n = 3) was examined at room temperature via spectrophotometry (723N, Changzhou, China) at 400–800 nm. Tensile strength was examined using stretch–stress tests [18]. Both ends of each sample (n = 5) were held with a clip and pulled vertically with a uniaxial stretching device until broken. The aCM (n = 3) and dAM (n = 3) were immersed in 35-mm diameter cell culture dishes and exposed to 0.25 mg/ml collagenase (Sigma) at 37°C for 60 min. Images were captured every 10 min.

### 2.5 In vitro and in vivo biocompatibility analysis

MTT analysis was performed to investigate biocompatibility in vitro. Briefly, the aCM conditioned medium was obtained by immersing 2 cm^2^ aCM in 1 ml normal culture medium (DMEM/F12 supplemented with 2% fetal bovine serum) for 24 h at 37°C. The normal medium served as a negative control. Subsequently, 100 µl (1×10^4^ cells/ml) of SV 40-immortalized human corneal epithelial cells (presented by Choun-Ki Joo of The Catholic University of Korea) was seeded into each well of a 96-well plate and incubated with 100% conditioned medium or fresh normal medium. At days 1, 3, 5, and 7, the proliferative activity of the cells was examined quantitatively using an MTT assay. Optical density (OD) was measured at 490 nm using a multifunctional microplate reader [19].

To investigate biocompatibility with corneal tissue in vivo, the aCM (3×4 mm) was implanted into intracorneal stroma (n = 3) utilizing a 4-mm-diameter pocket in the rabbit corneal stroma. One suture of 10–0 nylon was placed around the corneal wound. Corneal transparency and neovascularization were assessed via slit-lamp microscopy. One month later, the aCM-implanted cornea was stained with H.E. to examine the in vivo biocompatibility [20].

### 2.6 Construction of tissue-engineered corneal epithelium

Rabbit primary limbal epithelial cells were isolated and cultured as described previously, with minor modifications [21]. Rabbit limbal tissues were obtained by removing central corneas using an 8-mm-diameter trephine. The tissues were treated with 2.4 U/ml dispase for 1 h, followed by 0.25% trypsin/0.02% EDTA for 30 min at 37°C. The cell suspension (2×10^5^ cells per ml) was inoculated onto the spreading scaffolds of aCM and dAM. The samples were then examined via H.E. staining, immunofluorescence staining and ultrastructural analysis. For immunostaining, the constructed corneal epithelium were fixed with 4% paraformaldehyde for 20 min, incubated with the mouse anti-human monoclonal antibody K3/12 (1∶100, Chemicon, Temecula, CA) overnight at 4°C, and then incubated at 37°C for 30 min with TRITC-conjugated goat anti-mouse IgG (1∶200, Chemicon, Temecula, CA). Finally, the samples were stained with DAPI and examined via confocal microscopy (Nikon, Tokyo, Japan). Ultrastructure analysis was performed as a traditional preparation, and samples were observed via TEM. To track transplanted donor cells, the isolated rabbit corneal epithelial cells were labeled with CM-DiI (5 µg/ml, Invitrogen, Carlsbad, CA) according to the manufacturer's instructions.

### 2.7 Transplantation and evaluation of tissue-engineered corneal epithelium

#### 2.7.1 Construction and characterization of a rabbit limbal stem cell deficiency model

Twenty rabbit models with LSCD were constructed according to a previous report [22]. Rabbits were subjected to 2-mm-wide and 0.2-mm-deep limbal lamellar keratectomy, 10-mm diameter chemical trauma on the central corneal epithelium with n-heptanol (Alfa Aesar, Ward Hill, MA) and removal of a 3-mm-wide strip of conjunctiva close to the limbus. The rabbit LSCD model was characterized by impression cytology of the corneal epithelium, H.E. staining and two clinical indices (opacity and corneal neovascularization; Table 1) assessed through slit-lamp microscopy.

**Table 1:** Scoring for corneal surface.

item	corneal haze	neovascularization
0	no	no
1	slight, texture of iris is clear	within limbus and ≤2 mm
2	medium, texture of iris is dim	on peripheral cornea with extent ≤1/2 quadrant
3	severe, pupil is barely seen	on peripheral cornea with extent >1/2 quadrant
4	very severe, pupil is not seen	on whole cornea

#### 2.7.2 Transplantation of tissue-engineered corneal epithelium

Fifteen rabbits with LSCD were divided into three groups. Groups A and B underwent transplantation. This was achieved with tissue-engineered corneal epithelium, using aCM in group A and dAM in group B as scaffolds. In contrast, group C underwent transplantation with only aCM. Postoperatively, the transplanted corneal epithelium was evaluated according to the scores for corneal opacity and neovascularization, H.E. staining, corneal impression cytology and the existence of donor cells on the recipient corneal surface.

### 2.8 Statistical analysis

Student's t-test was employed for the statistical analysis of the physical characteristics of the conjunctiva matrix and MTT assay. Analysis of variance was used to evaluate the corneal opacity and neovascularization.

## Results

### 3.1 Decellularization efficiency

**Figure 1:**
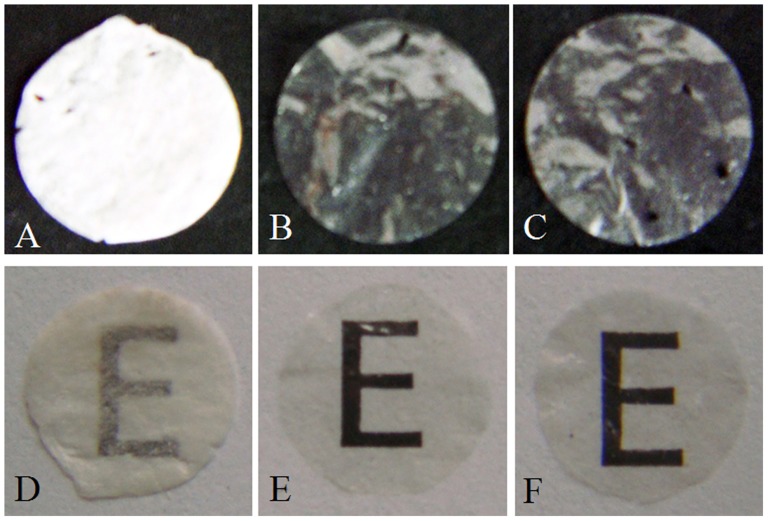
Macroscopic view of the acellular conjunctiva matrix. The aCM (B, E) were more transparent than the normal conjunctiva matrix (A, D). The transparent characteristics were not affected after the sterilization with γ-irradiation (C, F).

**Figure 2:**
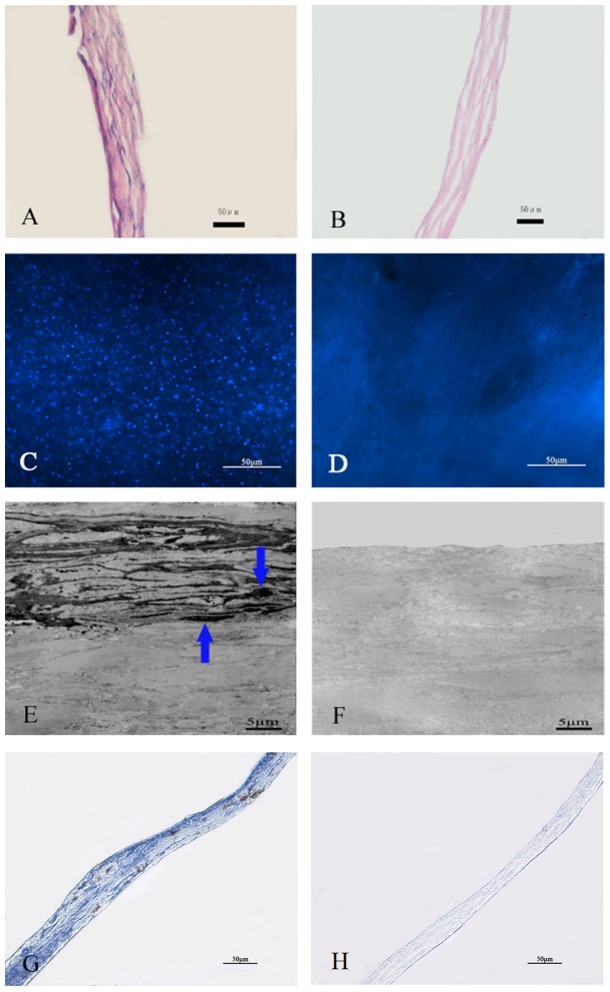
Efficiency of decellularization using 0.1% SDS. Native conjunctiva (A, C, E, G) maintained the typical epithelium and stroma morphology, whereas H.E. staining (A, B), Hochest staining (C, D), transmission electron microscopy (E, F) and immunohistochemistry (G, H) revealed no cellular component and α-gal in the aCM (B, D, F, H). Scale bar: 50 µm (A, B, C, D, G, H) and 5 µm (E, F).

The decellularized and sterilized conjunctiva matrix maintained its transparent characteristics during the prepared period (Fig. 1). Compared with the native conjunctiva, no intact cells were observed in the aCM with H.E. or Hochest staining (Fig. 2). Ultrastructural observation showed that the collagen fibrils of the aCM were tight and regular, with no cellular debris in the aCM (Fig. 2). The collagen fibrils of native conjunctiva were aligned slightly more tightly than were those of the aCM (Fig. 2). The α-gal detection showed that there was no α-gal antigen in the aCM compared with the large amount of α-gal staining in the native conjunctiva, which was visualized as brown particles (Fig. 2). The levels of DNA in the aCM and native conjunctiva were 1.01±0.12 µg/mg and 12.46±1.51 µg/mg dry weight, respectively, indicating that the DNA content of aCM was much lower than that of native conjunctiva (n = 5, p<0.05). The GAG levels in aCM and native conjunctiva were 48.63±3.40 µg/mg and 52.09±4.46 µg/mg, respectively; the hydroxyproline levels were 74.87±7.09 µg/mg and 82.07±4.95 µg/mg, respectively (n = 5, p>0.05).

### 3.2 Physical characteristics and stability of aCM

**Figure 3:**
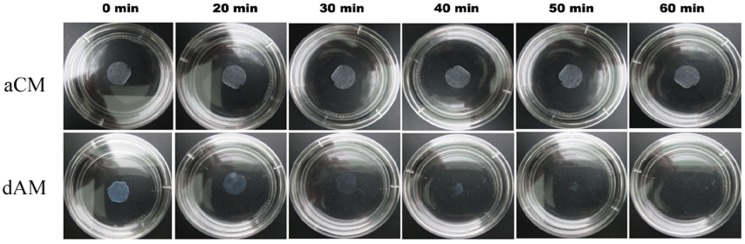
Morphological changes in acellular conjunctiva matrix and denuded amniotic membrane after 0.25% collagenase digestion.

The results of optical coherence tomography analysis showed that aCM was thicker than dAM (52.6±4.8 µm vs. 35.4±3.7 µm) (n = 5, p<0.05). However, there was no significant difference in optical transmittance between aCM and dAM (87.8±3.9% vs. 91.7±2.4%) (n = 3, p>0.05). The stretch–stress tests were performed on 10×30-mm samples to determine the maximum tear resistance. The results showed significant differences in tensile strength between aCM and dAM (7.9±0.6 gf vs. 5.8±0.5 gf) (n = 5, p<0.05) under wet conditions. The tensile elastic modulus of aCM and dAM was 23.6±3.4 and 14.3±2.1 MPa, respectively. In terms of stability, the dAM started to degenerate at 20 min and finally disappeared at 40–60 min (Fig. 3), whereas the aCM displayed no changes from 0–60 min (Fig. 3).

### 3.3 Biocompatibility in vitro and in vivo

**Figure 4:**
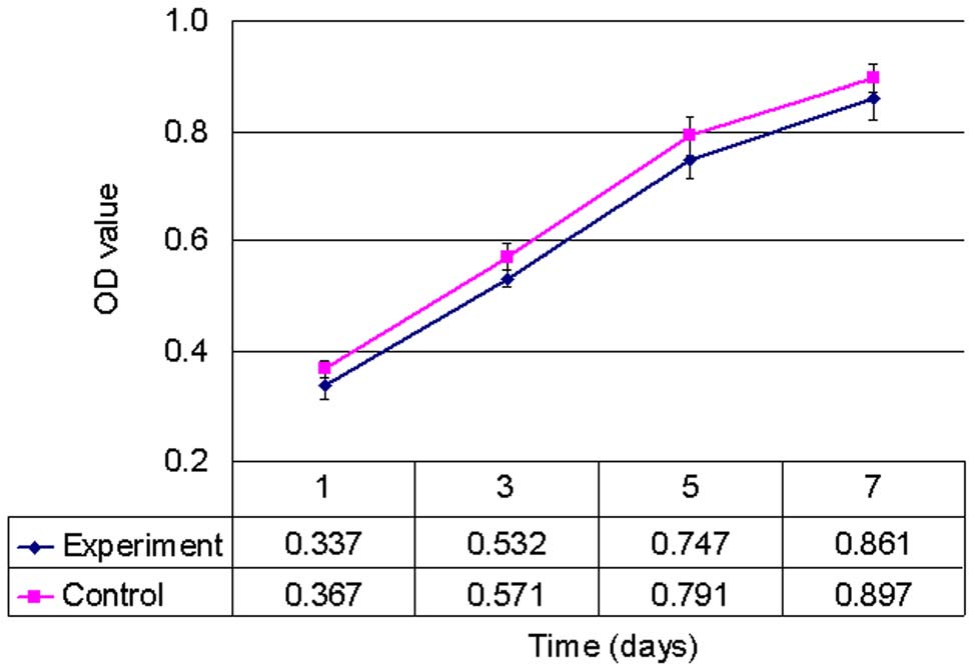
The effect of acellular conjunctiva matrix extracts on the proliferation of human corneal epithelial cells.

**Figure 5:**
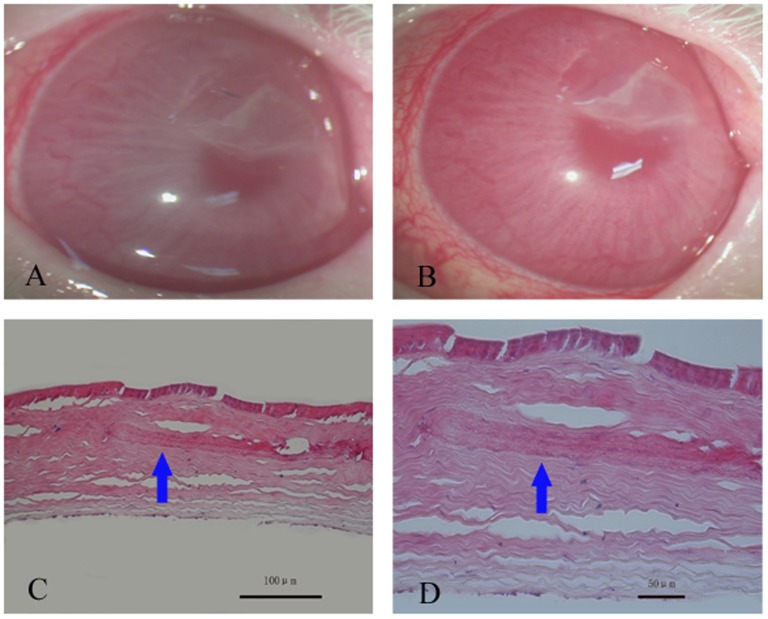
Biocompatibility of acellular conjunctiva matrix in vivo. Representative slit-lamp photographs just after intracorneal transplantation (A) and one month later (B). H.E. staining showed that transplanted aCM (arrow) adapted well to the host corneal stroma, with no evidence of inflammatory cells or stromal edema. Scale bar: 100 µm (C) and 50 µm (D).

The results of the MTT assay revealed no significant difference between the experimental and control groups (Fig. 4, n = 5, p>0.05), suggesting that aCM resulted in no significant cytotoxicity to the cultured corneal epithelial cells. Furthermore, there was no neovascularization on the rabbit cornea with aCM intracorneal transplantation (Fig. 5A, B), and the transplanted aCM integrated well into the host corneal stroma, with no evidence of inflammatory cells or edema (Fig. 5C, D).

### 3.4 Construction of tissue-engineered corneal epithelium in rabbit

**Figure 6:**
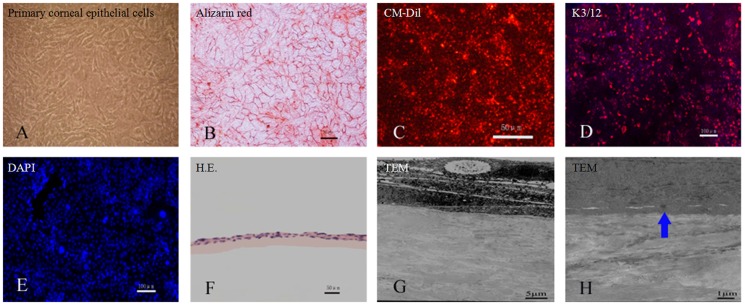
Construction of rabbit corneal epithelium tissue-engineered with acellular conjunctiva matrix. Rabbit primary limbal epithelial cells proliferated well at day 7 (A). Trypan blue-alizarin red staining showed that the cells maintained live activity and grew to confluence (B). The cells could be labeled with CM-Dil (red fluorescence) (C) and stained positive with anti-K3/12 (D). Negative control lacks primary antibody (E). The rabbit corneal epithelial cells formed a 2–3 epithelial layer structure after culture for 14 days, as confirmed by H.E. staining (F) and TEM observation (G). There were tight junctions between cells and the aCM scaffold (H). Scale bar: 100 µm (D, E), 50 µm (B, C, F), 5 µm (G) and 1 µm (H).

Rabbit primary corneal epithelial cells were labeled with CM-Dil and inoculated on the scaffolds of aCM and dAM. At day 7, the rabbit corneal epithelial cells proliferated well on both scaffolds (Fig. 6). Trypan blue-alizarin red staining showed that the cells were alive and grew to confluence on the scaffold (Fig. 6). K3/12 (markers of corneal epithelium) staining was positive (Fig. 6). At day 14, H.E. staining showed that the cells had formed a 2–3 epithelial layer structure (Fig. 6). TEM elucidated tight junctions between these cells and the aCM scaffold (Fig. 6).

### 3.5 Evaluation of tissue-engineered corneal epithelium

#### 3.5.1 Characterization of a rabbit model of limbal stem cell deficiency

**Figure 7:**
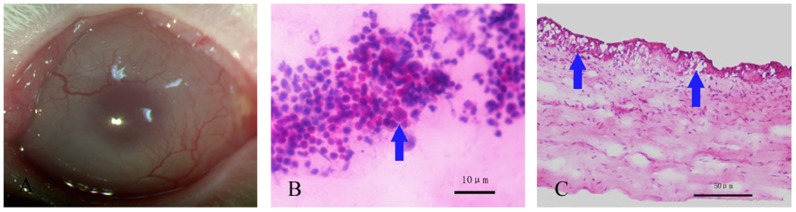
Construction of a rabbit limbal stem cell deficiency model. Representative slit-lamp photograph of rabbit cornea showed significant opacity and neovascularization after 1 month (A). PAS staining in which goblet cells are easily observed in corneal impression cytology (B). Signet ring goblet cells (arrow) were also revealed by H.E. staining of corneal sections (C). Scale bar: 10 µm (B) and 50 µm (C).

Corneal opacity and neovascularization could easily be observed one month postoperatively (Fig. 7A). Eighteen of the 20 rabbits scored ≥2 in corneal opacity and ≥3 in corneal neovascularization; imprint cytology revealed goblet cells with H.E. staining and positive periodic acid-shiff's (PAS) staining in corneas from two randomly selected rabbits (Fig. 7B, C).

#### 3.5.2 Clinical evaluation of transplanted corneal epithelium

**Figure 8:**
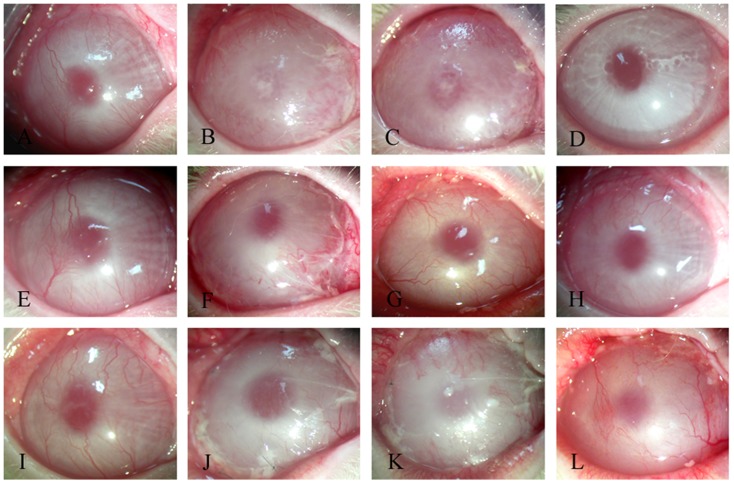
Clinical outcomes after transplantation of tissue-engineered corneal epithelium. Representative images of rabbit corneas transplanted with tissue-engineered corneal epithelium on aCM (A–D), tissue-engineered corneal epithelium on dAM (E–H), or aCM alone (I–L) at day 0 (A, E, I), day 7 (B, F, J), day 14 (C, G, K) and day 30 (D, H, L).

Postoperatively, there were no significant differences among the 3 groups until day 7. However, degradation of the transplanted dAM began at day 7 and continued until day 14, whereas the aCM began to degrade at day 21 and continued to degrade until day 28. Moreover, compared with the dAM-based reconstruction of the corneal epithelium, transplantation of the aCM-based reconstructed corneal epithelium more effectively reduced corneal haze and neovascularization; corneal opacity was restored completely at day 30 (Fig. 8). The differences in corneal epithelium scoring among all groups were not statistically significant at days 7 and 14 (p>0.05), whereas the differences among the three groups were statistically significant at day 30 (p<0.05) (Table 2).

**Table 2:** Scores of the corneal surface over time in all groups (corneal haze/neovascularization/sum).

Group	Day 7	Day 14	Day 30
A	2.0±0.3/2.4±0.2/4.4±1.1	1.6±0.4/2.2±0.4/3.5±1.3	0.4±0.2/0.6±0.2/1.1±0.3
B	2.4±0.5/1.8±0.2/4.2±1.1	1.6±0.2/1.8±0.4/3.3±1.3	1.2±0.2/1.4±0.2/2.5±0.6
C	2.2±0.4/2.4±0.5/4.0±1.2	2.2±0.4/2.4±0.5/4.2±1.1	1.8±0.4/3.2±0.4/4.6±0.9

#### 3.5.3 Corneal re-epithelialization and impression cytology analysis

**Figure 9:**
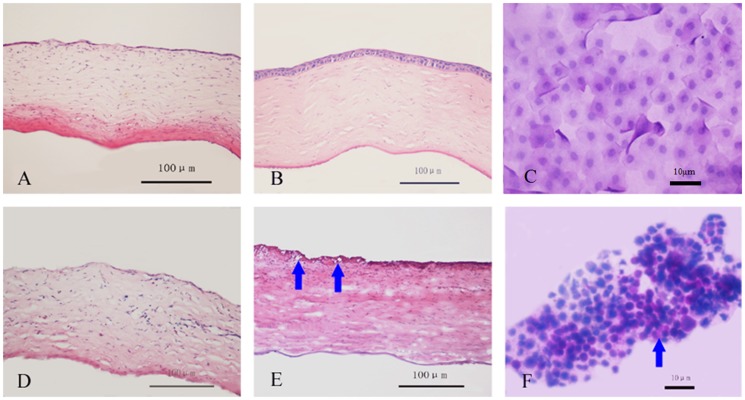
Corneal H and E staining and impression cytology after transplantation of tissue-engineered corneal epithelium. Restoration of corneal epithelium began at day 7 after transplantation with reconstructed corneal epithelium on aCM (A). Normal morphology was observed at day 30 (B) with no goblet cells (C), whereas the group that received reconstructed corneal epithelium on dAM exhibited defective corneal epithelium at day 7 (D) and goblet cells (arrow) were still observed at day 30 (E, F). Scale bar: 100 µm (A, B, D, E) and 10 µm (C, F).

**Figure 10:**
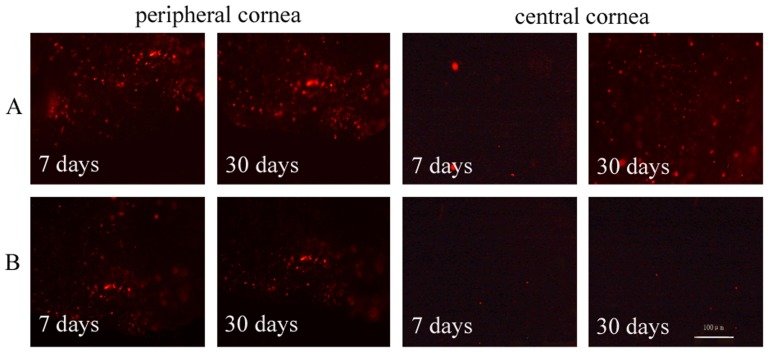
Postoperative tracking of donor cells on the recipient cornea. More donor cells were detected on peripheral region of the rabbit cornea transplanted with aCM (A) compared with that transplanted with dAM (B) at days 7 and 30. At day 30, more donor cells had migrated into the central cornea region transplanted with aCM-based corneal epithelium (A), whereas few cells were detected in the central cornea transplanted with dAM-based corneal epithelium (B). Scale bar: 100 µm.

Restoration of the corneal epithelium began at day 7 among rabbits that had been transplanted with aCM-based reconstructed corneal epithelium. Restoration was complete at day 30, with the appearance of normal corneal epithelium and no visible staining of goblet cells. In rabbit corneas transplanted with dAM-based reconstructed corneal epithelium, repair had not begun at day 7, and goblet cells could be observed until day 30 postoperatively (Fig. 9). Furthermore, more donor cells could be detected on the peripheral cornea transplanted with aCM-based reconstructed corneal epithelium compared with those transplanted with dAM-based epithelium at days 7 and 30. On the central cornea, there were more donor cells in the aCM group at day 30, although there were no differences between the groups at day 7 (Fig. 10).

## Discussion

The overall aim of this study was to develop and evaluate the aCM as a carrier of tissue-engineered corneal epithelium. In the present study, the decellularization method used to prepare aCM was first developed and optimized using 0.1% SDS for 12 h at 37°C. Next, the characteristics of the aCM, including DNA and GAG content, thickness, optical transmittance, tensile strength, ability to resist collagenase degradation, ultrastructural analysis, and cellular and tissue biocompatibility, were investigated extensively. Finally, we evaluated the effectiveness of using aCM as a carrier of tissue-engineered corneal epithelium in the rabbit model of LSCD.

The main index used to evaluate the efficiency of decellularization was the removal of donor DNA and the maintenance of extracellular matrix. Several detergents, such as SDS and Triton X-100, have been used in the decellularization of various natural materials [23]. In this study, we used SDS, an ionic detergent that binds to cell membranes by penetrating the bilayer and ultimately causing its dissolution. This technique removes the cellular components in porcine conjunctiva after the removal of Tenon's capsule. Compared with other detergents, SDS led to more complete removal of nuclear remnants and cytoplasmic proteins [24] while tending to disrupt the native tissue structure, resulting in reduced GAG content and a loss of collagen integrity. Hence, it is critical to control the SDS concentration and treatment time according to the origin and characteristics of the target tissue.

In preliminary studies, we found that treatment with 0.1% SDS for 24 h could remove 98% of DNA content but reduced the GAG content to 60% of the normal levels. When using 0.5% SDS for 12 h, the DNA content was reduced by 99%, and the GAG content was reduced to 50% of the normal levels. Finally, we used 0.1% SDS for 12 h for the decellularization of porcine conjunctiva. Compared with native conjunctiva, aCM exhibited a 92% drop in DNA content, whereas more than 90% of the GAG and collagen were retained. Although we did not detect residual SDS in aCM, an MTT assay of the aCM showed no cytotoxicity to corneal epithelial cells, indicating that the decellularization method was favorable because it removed most of the DNA components, retained more extracellular matrix and resulted in less residual SDS.

Bioengineered cornea has potential for clinical applications, particularly as a scaffold. At present, several synthetic and natural biomaterials have been investigated as potential scaffolds for tissue-engineered cornea. Natural biomaterials are more promising than synthetic biomaterials because of their flexibility and suitable physical and mechanical properties [23]. The aCM scaffold demonstrated physical characteristics that were appropriate for clinical applications, including thickness (52.6±4.8 µm), optical transmittance (87.8±3.9%), tensile strength (7.9±0.6 gf), and tight and regular collagen fibrils. Furthermore, aCM demonstrated few adverse effects on the proliferation of corneal epithelial cells and supported a multilayer structure and tight junction (desmosome) formation. Following intracorneal transplantation, aCM demonstrated favorable biocompatibility, with no evidence of inflammation or stroma edema at the injected site. To eliminate the possibility of viral contamination of the porcine conjunctiva, we used γ-irradiation to sterilize the scaffold, a method that could be employed in future clinical applications [23].

Compared with dAM, the aCM demonstrated comparable thickness, optical transmittance, tensile strength and biocompatibility. Additionally, the aCM was better than dAM in resisting collagenase degradation, suggesting that aCM may be more useful in the therapy of patients with severe ocular disorders. Moreover, we observed the outcomes of using these materials as scaffolds for tissue-engineered corneal epithelium for ocular surface reconstruction in the rabbit model of LSCD. There was no significant difference between the aCM- and dAM-based reconstructed corneal epithelium in the early phase (7–14 days). However, at day 30, the therapeutic effects of aCM-based reconstructed corneal epithelium were more stable than those of dAM-based corneal epithelium. Furthermore, we found that the degradation of dAM began earlier than did that of aCM. Additionally, the number of donor cells on the recipient cornea at day 30 was higher when using aCM compared with dAM as the scaffold, revealing that aCM retained donor cells longer in vivo compared with dAM. The prolonged survival of donor cells may be the key factor contributing to an improved therapeutic effect of aCM. In conclusion, the transplantation of tissue-engineered corneal epithelium using aCM as a scaffold can be employed successfully for reconstructing the ocular surface in rabbits with LSCD.

## Conclusions

We have presented a method of utilizing SDS to prepare xenogeneic aCM. The prepared aCM exhibited favorable biocompatibility, tensile strength, stability and transparency. This material can be used as a scaffold to tissue-engineer corneal epithelium to reconstruct the ocular surface.
